# Modeling the future of cancer registration and research: The Martinique Cancer Data Hub Platform

**DOI:** 10.7189/jogh.10.020352

**Published:** 2020-12

**Authors:** Clarisse Joachim, Mylène Vestris, Miguelle Marous, Thierry Almont, Stephen Ulric-Gervaise, Moustapha Dramé, Cédric Contaret, Juliette Smith-Ravin, Patrick Escarmant, Emmanuelle Sylvestre, Jacqueline Véronique-Baudin

**Affiliations:** 1 CHU de Martinique, Pôle de Cancérologie Hématologie Urologie, Registre Général des cancers de la Martinique, Martinique, France; 2 CHU de Martinique, Pôle de Cancérologie Hématologie Urologie, Martinique Cancer Data Hub, Martinique, France; 3 CHU de Martinique, Pôle de Cancérologie Hématologie Urologie, Recherche en Cancérologie Hématologie, Martinique, France; 4 CHU de Martinique, Délégation de la Recherche et de l’innovation, Martinique, France; 5 Université des Antilles, Groupe de recherche BIOSPHERES, Campus de Schœlcher, Martinique, France; 6 CHU de Martinique, Pôle de Cancérologie Hématologie Urologie, Martinique, France; 7 INSERM, Rennes, France; 8 Université de Rennes 1, LTSI, Rennes, France; 9 CHU de Martinique, Department of Infectious Diseases, Martinique, France

The number of Clinical Data Research Networks (CDRN) focused on creating large collections of data from multiple digital sources, has vastly increased in the last few years. These CDRN are particularly relevant in outlying island regions, where they make it possible for those involved to communicate and contribute, thereby facilitating remote collaborations, despite the physical distance.

We propose an innovative and robust organizational networking platform with advanced digital tools and strong partners, to boost a collaborative medical network of Public Health and Health Surveillance, in close connection with clinical research, and population-based cancer registries. A multifunctional Information and Communication Technology (ICT) platform is proposed. This platform will provide institutions and health care professionals with a “Cancer Data Hub”, which will offer a wide range of accurate cancer data (patterns of cancer care, quality of life, onco-pharmaco-epidemiology…), fostering collaborative and innovative public health research activities (eg, observational studies in real life practice and clinical trials).

Blockchain modelization will be adapted with interconnected work-packages in the area of cancer. A completely secure environment, with powerful, simplified interfaces for software and hardware will be created, with interoperability of interfaces, databases managed in Big-Data, e-learning, research and a social network interface for online access. Development of innovative electronic tools is also ongoing for patient-reported outcomes. This platform addresses the challenges that need to be resolved in the Caribbean area, with a view to improving overall quality of life and survival, by increasing capacity to implement telemedicine technologies and health care tools, also with a focus on research.

Population-based cancer registries (PBCRs) participate in epidemiological surveillance and in the evaluation of cancer types worldwide by enabling analysis of incidence and survival data over time. Thanks to continuous, exhaustive recording of all cancer diagnoses, and follow-up of all cases until death occurs, studies of cancer incidence and survival data can be performed to describe the spatio-temporal distribution of cancer localisations, and to evaluate the efficacy of management by the health care system [1,2]. Not all countries in the Caribbean have population-based cancer registries to evaluate the extent of disease [3,4]. It would therefore be beneficial if other, existing registries could contribute to producing international epidemiological statistics for this area. Cooperation among the islands of the Caribbean, and with the Latin-American zone would make it possible to meet these objectives with the support of the International Agency for Research development and Global initiative for cancer registry development worldwide [5,6].

Martinique, a French West Indies overseas territory, has an exceptional heritage of reliable cancer data since 1983, thanks to the Martinique Cancer Registry. This registry is a high-quality PBCR classified among the French overseas territories, and officially certified by the French Registry Evaluation Committee (Comité d’Evaluation des Registres). This committee awarded our Registry the top grade (grade A – Excellent) for the quality of its data, its expertise in public health, and for the quality of its research at national and international levels [7].

Clinical Data Research Networks are becoming increasingly widespread because of their ability to offer a collaborative environment for researchers localized across disparate organizations [8,9]. The PCORnet initiative launched in the United States in 2014 [8] is one of the most prominent examples. The aim of these networks is to support effective and sustainable research infrastructures based on electronic health data, and to facilitate multi-site community-engaged research.

Although CDRN have become an integral part of research practice in the USA, heralding a paradigm shift regarding the use of Electronic Health Records (EHR) for research [10], there are still very few such CDRN outside of the USA, and none in the Caribbean region, despite the geographical proximity. This is why the implementation of the “Martinique Cancer Data Hub” is innovative and unique in these territories. This project is an opportunity to develop collaborative programs with patients, patient associations and health professionals. This challenge will consolidate existing experience, in close connection with the competence and cooperation of PBCRs, and in collaboration with other stakeholders in the cancer information domain. This process can be supported by the mobilisation of external knowledge initiatives, such as professional societies, collaborative research partnerships and implementation networks (International Agency for Research on cancer – IARC, International Association of cancer registries – IACR) [11].

## DELIVERABLES

We designed the platform concept and content in iterative cycles, with a bottom-to-top approach, by consulting future users from different backgrounds (scientists, clinicians, patients…), experts and project partners, and finally software developers. Various developments in terms of database organization and security are planned, in accordance with internationally recognized standards.

The platform will also have an e-learning component with all published articles of the partners and Massive Open Online Courses (MOOC). Access to the platform will be carefully controlled by a secure research portal with full traceability features. Finally, a social network interface will allow patients and health professionals to engage with each other. We will also propose access to open databases and evaluation interfaces.

**Figure 1:**
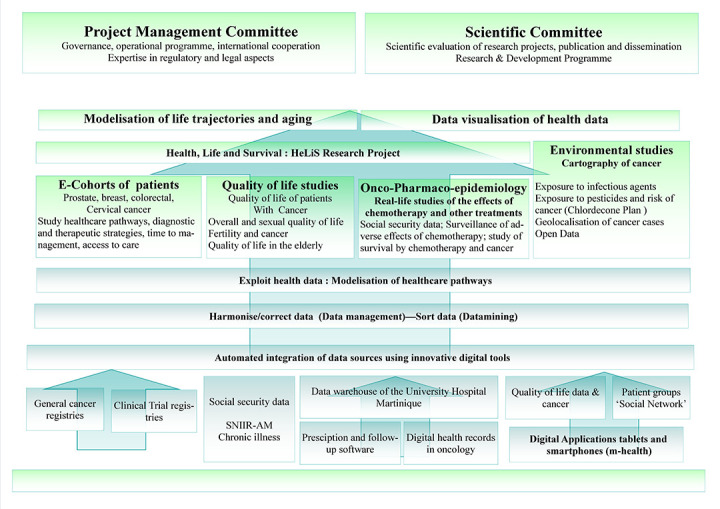
Strategic plan for cancer research and development of the Martinique Cancer Data Hub.

The Martinique Cancer Data Hub will propose interactive digital tools for data-mining and data-visualization of health data, digital training tools (e-learning), digital tools of m-health (health via smartphones and connected objects) and modeling of life and aging trajectories (**Figure 1**). This secure internet platform should enable health professionals to better evaluate health care pathways through modeling of health and data visualization, thanks to a laboratory generated for the exploitation of health data in oncology.

The proposed solution will allow geographically dispersed teams to leverage clinical and biological information, enabling all participants to view and share on any device, the flexible and interactive content of each conversation field, in order to increase efficiency and productivity.

The collaborations set up will make it possible to avail of comparative data to initiate research on topics such as cancer inequalities, surveillance of infectious cancers, and role of environmental factors or specific exposures in our geographical area, and to identify the determinants of health states in cancer patients in Martinique. Ultimately, the Martinique Cancer Data Hub platform should facilitate monitoring and data collection as part of population monitoring.

Establishing legal frameworks for health technology innovations is thus, in a context of highly sensitive data, the preliminary step to initiating large-scale projects within the framework of potential multicenter projects.

The e-learning and e-health platform, developed in close collaboration with the Clinical Data Warehouse project of the University Hospital of Martinique, is an innovative and polyvalent technology. Expertise in training in the area of public health will increase the attractiveness and the opportunities for collaboration in an environment where the mutualisation of means and resources is strongly encouraged.

In a second stage, this federating project could be extended to other countries participating in cancer surveillance.

This challenge will increase the number of innovative digital products including the transfer of technologies in the field of “e-health”, including the axes of aging. Public health actions will therefore have to be implemented in the coming years, given the foreseeable impact of aging on the demographic and health profile.

This platform will represent a strategy dedicated to the emergence of new research topics with high economic potential, for the benefit of patients, medical communities and health care actors.

## HEALTH LIFE AND SURVIVAL IN THE ELDERLY: THE HeLiS PROJECT

As an example of a project involving patients, we propose to invite senior citizens to join an online community with a view to creating an online “living space”, a forum for exchange of ideas and practices oriented towards improving quality of life in older persons. The participants in this initiative will have access to quality of life research projects thanks to the development of a social network integrating older persons, patient communities and caregivers. The aim is to constitute a group of older experts who will engage in quality of life initiatives for seniors.

**Figure:**
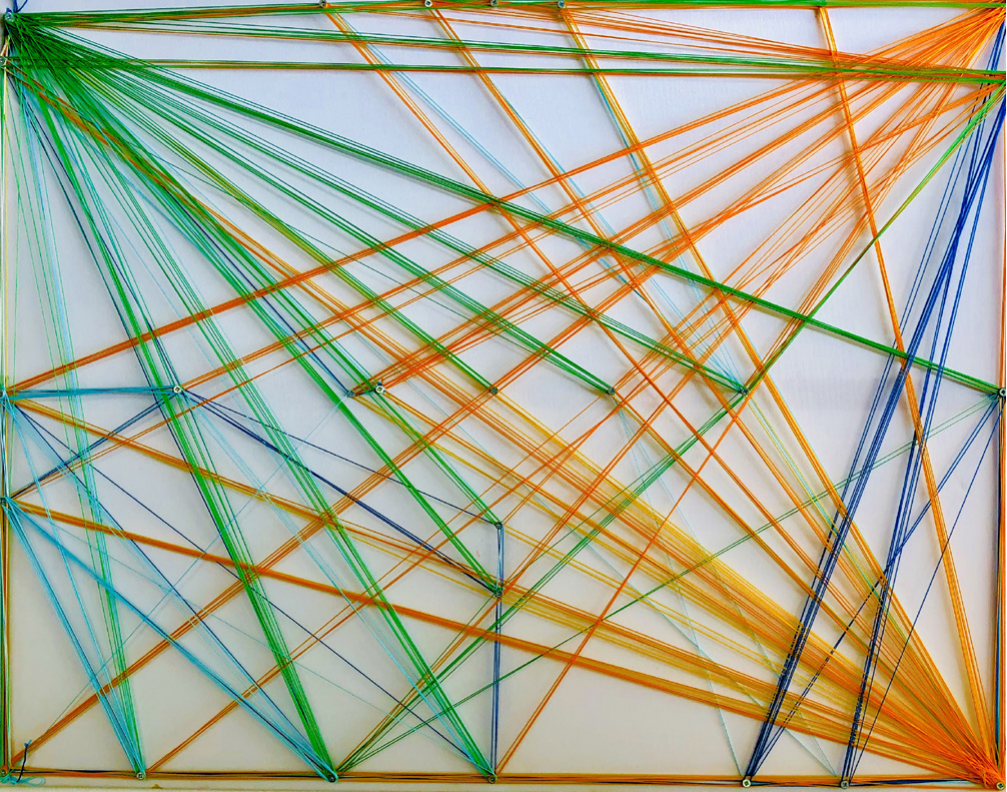
Photo: Allegory of the Martinique cancer data Hub: an arborescent map of visualizing cancer dataflow (from the collection of Clarisse Joachim, used with permission).

This project meets several different public health goals, namely public health plans targeting successful aging, and solidarity with older citizens. It will promote a life-course prevention approach to achieve successful aging, and is notably in line with actions targeting the physical health and physical environment of older people, with local and environmental participative activities, in addition to maintaining social links. HeLiS is a programme with potential public health benefit in the quality of life domain, specifically dedicated to older persons in Martinique. It has strong potential to modelize life trajectories and aging pathways, thanks to longitudinal analysis of quality of life data. The impact of preventive measures and physical activity on quality of life is key to evaluating health status in older persons in Martinique via the development of e-health. Other similar ongoing projects will be accessible to health care professionals, and to patients who desire to participate in research. A study of the quality of life of patients with prostate cancer is currently ongoing in the University Hospital of Martinique.

### Cohort profile: the Martinique Cancer Registry and the quality of life in prostate cancer cohort (QoL Prostate-MQ): Challenges and prospects for reducing disparities in the Caribbean

The quality of life in prostate cancer (PCa) cohort will assess quality of life and patient outcomes in Martinique using the digital platform for patient-reported outcome measures. This study will provide insights into the high rates of incidence and survival of PCa observed in Martinique, as compared with other Caribbean countries. The quality of life PCa cohort will be constituted in the context of our established and internationally recognized cancer registry, thus promoting high-quality data verification and recording. Data on clinical stage at diagnosis, blood prostate-specific antigen level at diagnosis, Gleason score, primary treatment, quality of life, urinary incontinence and erectile dysfunction prior to treatment will be analysed, where applicable, at 1 and 5 years after treatment.

### Combining electronic quality of life surveys, reimbursement claims databases and Clinical Data Warehouse to improve adverse drug reaction detection in breast cancer treatment in Martinique

There were 205 incident cases of breast cancer per year in Martinique from 2007 to 2014. We will conduct a prospective study including female patients who start treatment for breast cancer at Martinique University Hospital between 2020 and 2022. The main aim of the study is to detect adverse drug reactions related to breast cancer treatments in Martinique, using two types of sources: 1) Patient Reported Outcomes from two Quality of Life (QoL) questionnaires and 2) Clinical reported outcomes from two databases, namely the French Nationwide Claims and Hospitalization Database (Système National des Données de Santé, SNDS) and the clinical data ware house used at Martinique University Hospital.QoL will be measured using the European Organisation for Research and Treatment of Cancer Quality of Life Questionnaire Core 30 (EORTC QLQ-C30) and the Breast cancer module QLQ-BR23, which have both been validated for completion via electronic means. The questionnaires will be administered by the caregiving team, or may also be self-completed by the patient via an online link to the digital platform for data collection and quality control. This will ensure the exhaustiveness of the variables included in the database, by using data quality control programmes to verify that these data are collected systematically.

### E-learning and cancer registration: developing tools for data knowledge and reporting

Available training modules will be integrated into the online digital platform, focusing on the epidemiology of cancer, the study of cancer survival according to identifiable risk factors, and methods for data collection from digital sources. This new approach to cancer registration will be based on the ability to extract data from the clinical data warehouse project at the University Hospital of Martinique. The first phase of learning will cover data extraction models, and this will serve as a basis for the development of data integration algorithms for individual projects. This project will reveal the potential of new information and communication technologies to cross-reference nominative data directly for the purposes of cancer surveillance. All the training modules will be available online in French, English and Spanish, to enable maximum interaction and exchange with other countries in the Caribbean and in Latin-America.

### Main barriers to development

Among the challenges of implementing digital projects in the field of public health, access to digital patient files is a major issue. Indeed, one of the attractions of the digital platform is to enable cross-referencing and analysis of digitally processed data. The platform is therefore logically limited by the available of digital source data within the sites of health care delivery. Indeed, identifying gaps in the information technology and digitalization of the sites of care is one of the aims of the project, so that we can identify the specific types of data that need to be processed digitally, and prepare a roadmap for going fully digital, using appropriate software.

The year 2020 saw a pandemic of COVID-19 that called on health care teams worldwide to make rapid, yet profound adjustments to their working environment to introduce telemedicine and tele-reporting features at short notice. The health care system should have the capacity to deal with unprecedented situations that require rapid implementation of e-health solutions to record health data during a health care crisis.

Finally, in terms of research, another major challenge of our digital platform is that one needs to take into consideration the age of the patients, and how this may affect their understanding of, and ability to use the digital tools that are made available to them. Indeed, paper forms remain the preferred format for many people, depending on their age. In addition, the material and social conditions of the patients included also deserve consideration. Digital processing of data collected in paper format is feasible, but requires research teams to allocate time and human resources for this task. Furthermore, the quality of the internet connection, and the provision of access in the island context are also important factors to consider, in order to ensure access for the widest possible audience.

## INTERNATIONAL MODELS OF DATA PLATFORMS

Digital data platforms are garnering increasing interest around the world, for the purposes of collaboration, research and dissemination of knowledge.

The French “ComPaRe” project (https://compare.aphp.fr/) (COMmunity of PAtients for REsearch)is a prime example of patients and researchers coming together. This model of publicising ongoing research and disseminating knowledge on various topics is a rich source of data and of scientific output. Another study platform for comparative outcome, quality-of-life, and translational research for genitourinary cancer was created in Seoul, an supports a variety of research regarding comparative outcomes, QoL studies, and translational research [12]. In a collaborative project bringing together Denmark, Norway, Portugal, Spain, Sweden and the UK, *I-O Optimise* is a multinational program providing real-world insights into lung cancer management. This project will provide a broad, robust and dynamic research platform to continually address numerous research objectives in the lung cancer arena from more than 45 000 patients per year [13]. Finally, the Korea Cancer Big Data Platform (K-CBP) is a further example of a multi-database framework that collects clinical, genomic, imaging, and biobank data for cancer research [14]. Currently, there is no platform that includes real-time input of research data in patients with cancer in the French West Indies. Our project will meet this need, and demonstrate that e-health solutions provide a unique opportunity to reduce inequalities in outcomes and care among cancer patients, in line with the public health objectives of the French national anti-cancer plan.

## CONCLUSION

This project will enhance partnership in the fight against cancer, using patient and clinical-reported outcome measures. Future plans, beyond the present cohort, aim to pursue this research work as a tool to inform policy in the areas of population health and health services research. We hope that using e-health tools such as online surveys and the clinical data warehouse will help to improve the standards of care for cancer treatment in our overseas territories, despite the remote location.Shared needs in terms of public health include cancer surveillance and public health surveillance; and the evaluation of public health programmes in cancer. The needs in terms of scientific research are similar across the region, despite the fact that the socio-economic level of the French West Indies is higher than that of the majority of other countries in the Caribbean. The role of national and regional decision-makers is clearly essential: strong political enthusiasm is a *sine qua non* and will always be the key prerequisite for the success of this platform.

## References

[1] Bray F, Znaor A, Cueva P. Planning and developing population-based cancer registration in low- and middle-income settings, IARC Technical Publications; 43: International Agency for Research on Cancer. 33502836

[2] Parkin DM The role of cancer registries in cancer control. Int J Clin Oncol. 2008;13:102-11. 10.1007/s10147-008-0762-6 18463952

[3] Razzaghi H Quesnel-Crooks S Sherman R Joseph R Kohler B Andall-Brereton G Leading Causes of Cancer Mortality - Caribbean Region, 2003-2013. MMWR Morb Mortal Wkly Rep. 2016;65:1395-400. 10.15585/mmwr.mm6549a3 27977639

[4] Bray F Ferlay J Soerjomataram I Siegel RL Torre LA Jemal A Global cancer statistics 2018: GLOBOCAN estimates of incidence and mortality worldwide for 36 cancers in 185 countries. CA Cancer J Clin. 2018;68:394-424. 10.3322/caac.21492 30207593

[5] Goss PE Lee BL Badovinac-Crnjevic T Strasser-Weippl K Chavarri-Guerra Y St Louis J Planning cancer control in Latin America and the Caribbean. Lancet Oncol. 2013;14:391-436. 10.1016/S1470-2045(13)70048-2 23628188

[6] Strasser-Weippl K Chavarri-Guerra Y Villarreal-Garza C Bychkovsky BL Debiasi M Liedke PE Progress and remaining challenges for cancer control in Latin America and the Caribbean. Lancet Oncol. 2015;16:1405-38. 10.1016/S1470-2045(15)00218-1 26522157

[7] Joachim C Veronique-Baudin J Ulric-Gervaise S Pomier A Pierre-Louis A Vestris M Cancer burden in the Caribbean: an overview of the Martinique Cancer Registry profile. BMC Cancer. 2019;19:239. 10.1186/s12885-019-5434-6 30876409 PMC6420743

[8] Fleurence RL Curtis LH Califf RM Platt R Selby JV Brown JS Launching PCORnet, a national patient-centered clinical research network. J Am Med Inform Assoc. 2014;21:578-82. 10.1136/amiajnl-2014-002747 24821743 PMC4078292

[9] Yuan J Malin B Modave F Guo Y Hogan WR Shenkman E Towards a privacy preserving cohort discovery framework for clinical research networks. J Biomed Inform. 2017;66:42-51. 10.1016/j.jbi.2016.12.008 28007583 PMC5316314

[10] Rosenbloom ST Harris P Pulley J Basford M Grant J DuBuisson A The Mid-South clinical Data Research Network. J Am Med Inform Assoc. 2014;21:627-32. 10.1136/amiajnl-2014-002745 24821742 PMC4078290

[11] IARC C-. IARC Caribbean Cancer Registry Hub 2018. Available: http://caribbeancrh.carpha.org/. Accessed: 20 June 2020.

[12] Jeong CW Suh J Yuk HD Tae BS Kim M Keam B Establishment of the Seoul National University Prospectively Enrolled Registry for Genitourinary Cancer (SUPER-GUC): A prospective, multidisciplinary, bio-bank linked cohort and research platform. Investig Clin Urol. 2019;60:235-43. 10.4111/icu.2019.60.4.235 31294132 PMC6607078

[13] Ekman S Griesinger F Baas P Chao D Chouaid C O’Donnell JC I-O Optimise: a novel multinational real-world research platform in thoracic malignancies. Future Oncol. 2019;15:1551-63. 10.2217/fon-2019-0025 30852916

[14] Cha HS Jung JM Shin SY Jang YM Park P Lee JW The Korea Cancer Big Data Platform (K-CBP) for Cancer Research. Int J Environ Res Public Health. 2019;16:2290. 10.3390/ijerph16132290 31261630 PMC6651426

